# Review of the analysis of ^234^Th in small volume (2–4 L) seawater samples: improvements and recommendations

**DOI:** 10.1007/s10967-021-07772-2

**Published:** 2021-06-24

**Authors:** Samantha J. Clevenger, Claudia R. Benitez-Nelson, Jessica Drysdale, Steven Pike, Viena Puigcorbé, Ken O. Buesseler

**Affiliations:** 1 Department of Marine Chemistry & Geochemistry, Woods Hole Oceanographic Institution, 360 Woods Hole Rd, Woods Hole, MA 02543 USA; 2 Department of Earth, Atmospheric and Planetary Sciences, Massachusetts Institute of Technology, 77 Massachusetts Ave, Cambridge, MA 02139 USA; 3 School of the Earth, Ocean & Environment, University of South Carolina, 701 Sumter St, Columbia, SC 29208 USA; 4 School of Science, Centre for Marine Ecosystems Research, Edith Cowan University, 270 Joondalup Dr, Joondalup, WA, 6027 Australia

**Keywords:** Thorium-234 analysis, ^234^Th particle flux, Biological carbon pump, Particle scavenging

## Abstract

The short-lived radionuclide ^234^Th is widely used to study particle scavenging and transport from the upper ocean to deeper waters. This manuscript optimizes, reviews and validates the collection, processing and analyses of total ^234^Th in seawater and suggests areas of further improvements. The standard ^234^Th protocol method consists of scavenging ^234^Th from seawater via a MnO_2_ precipitate, beta counting, and using chemical recoveries determined by adding ^230^Th. The revised protocol decreases sample volumes to 2 L, shortens wait times between steps, and simplifies the chemical recovery process, expanding the ability to more rapidly and safely apply the ^234^Th method.

## Introduction

Thorium-234 (*t*_1/2_ = 24.1 days) is used to examine particle scavenging, residence times, and particle export at high spatial and temporal resolution, providing insights into the biogeochemical cycling of carbon (C), nutrients, trace elements, and pollutants in the ocean. The ^234^Th approach to measuring sinking particle export is based on the disequilibrium between the particle reactive radiogenic decay product ^234^Th, and its soluble parent, ^238^U (*t*_1/2_ = 4.5 × 10^9^ years). In oxygenated seawater, ^238^U exists predominantly as dissolved UO_2_(CO_3_)_3_^4−^, with ≤ 0.1% of ^238^U scavenged onto particles [1]. The radioactive progeny ^234^Th exists in seawater as Th(OH)_n_^(4−*n*)+^ [1, 2]. When no particles are present in seawater, ^238^U and ^234^Th are in secular equilibrium, meaning activities are equal. When particles are present, such as those resulting from biological activity, ^234^Th is rapidly scavenged (particle-water partition coefficient, *K*_*d*_ = 10^5^ cm^3^ g^−1^; [3, 4]). When those particles sink, they preferentially remove ^234^Th from the water column, causing disequilibrium with ^238^U. The deficit of ^234^Th (^234^Th < ^238^U) can therefore be used to measure the net flux of ^234^Th on sinking particles out of a given depth interval. The ^234^Th flux is subsequently used to derive a C flux by multiplying a known C:^234^Th ratio of sinking particles, often collected by sediment traps or in situ pumps [e.g., 5–8]. This approach has since been expanded to other elements and compounds, such as particle reactive organic contaminants and trace metals [e.g., 9–13].

^234^Th has been used as a particle scavenging tracer since as early as the late 1960’s, when Bhat et al. [14] used iron oxide (Fe(OH)_3_) to scavenge and concentrate ^234^Th from seawater, finding an increase in scavenging and export from open to coastal waters. Since then, the application of ^234^Th has become increasingly more widespread, especially after its application to quantifying C fluxes in the early 1990’s [15–22]. Methods of collection and analysis of ^234^Th in seawater have undergone significant changes. Early studies involved formation of a Fe(OH)_3_ precipitate, extensive anion-exchange purification to separate thorium from uranium and other beta emitters, and electroplating thorium onto metal planchets prior to beta counting. Other isotopes of thorium, first ^228^Th and then ^229^Th and ^230^Th, were used to trace recoveries resulting from losses associated with radiochemical processing, and allowed for a more accurate determination of ^234^Th activities [2, 14, 23]. Further development of the method led to ^234^Th analysis at sea via non-destructive gamma counting using large volumes (> 1000 L) of water pumped through manganese dioxide (MnO_2_) cartridges [e.g., 15, 18]. This reduced the need for chemical purification since uranium was separated from thorium during the scavenging step and ^234^Th is quantified uniquely by its gamma emissions at 63 keV. Rutgers van der Loeff and Moore [24] developed the MnO_2_ coprecipitation method using 20 L samples followed by direct beta counting that allowed for higher resolution sampling. Improvements in the method allowed for use of volumes as low as 2–4 L [e.g., 25]. Pike et al. [26] refined the protocol by adding ^230^Th as a yield monitor to correct for sample losses throughout processing. A history of ^238^U-^234^Th disequilibrium applications for ocean sciences are described in Cochran and Masque [27], and reviews of ^234^Th use specifically are available in Waples et al. [28], and Le Moigne et al. [29].

### Revised 2 L method overview

**Fig. 1:**
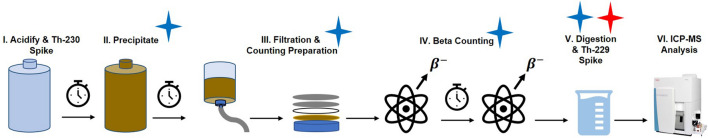
Schematic of the 2 L protocol presented in this manuscript. Data obtained from experiments unique to this manuscript are denoted with blue symbols, while changes that were carried out by other groups but presented here as part of this review are denoted with red symbols

The 2 L total ^234^Th method discussed here is adapted largely from methods described in previous work [e.g., 25–26, 30]. A 2 L sample volume was originally suggested by Benitez-Nelson et al. [25], but 4 L sample sizes continued to be popular because of the lower beta counting time required with larger volumes. The 2 L method detailed here, however, has proven to produce similarly high-quality results [31], and is manageable when sufficient beta counting capacity is available at-sea or on-land shortly after collection. This 2 L method is based upon the formation of a MnO_2_ precipitate (Fig. 1). Briefly, unfiltered 2 L water samples are collected, immediately acidified, and a ^230^Th yield monitor is added. Samples are allowed to equilibrate, pH is raised and potassium permanganate (KMnO_4_) and manganese chloride (MnCl_2_) are added to create a MnO_2_ precipitate. Once the precipitate fully forms, samples are filtered, dried and then counted twice using an anti-coincidence, 5 sample, low-level Risø beta counter. Samples are then allowed to decay for at least 6 half-lives (144 days) before a final beta count that quantifies the non-^234^Th background activities. Filters are then digested, spiked with ^229^Th as a secondary yield monitor, and analyzed directly by inductively coupled plasma mass spectrometry (ICP-MS) to correct for chemical recovery of the entire process based upon the initial ^230^Th added. This is a significant difference from the original method developed by Pike et al. [26] as it does not include anion exchange purification before ICP-MS measurement and removes the use of the hydrofluoric acid (HF), one of the most dangerous inorganic acids known.

This manuscript is organized following the steps shown in Fig. 1. Some of the steps include novel experiments conducted to improve upon the protocols of the Benitez-Nelson et al. [25] and Pike et al. [26] and to validate the use of 2 L samples (steps I, II, III, IV and V from Fig. 1).

## Updated method: Results and discussion for method improvements

### Step I: Sample collection and ^230^Th yield monitor

The sample collection protocol remains largely unchanged, except for the reduction in sample volume, motivated primarily by a want for more efficient sample collection and processing at sea. For open ocean samples, 2 L of unfiltered water is collected into 2 L fluorinated polyethylene (FPE) Teflon-coated bottles. Bottles are marked with a fill line that allows approximately 20 mL of headspace and pre-calibrated by weight for a more precise volume estimate. Sample volumes of 2 L are sufficient when the time period between collection and analysis is less than 1 week, otherwise, a larger volume may be needed to obtain a sufficient beta count signal. After collection, water is acidified to pH of 1–2 with ~ 3.5 mL concentrated nitric acid (HNO_3_). A known volume of ^230^Th (1 mL by weight, ~ 50 dpm g^−1^; or well above the ICP-MS detection limit) is added to serve as a yield monitor. Given the highly particle reactive nature of thorium, it may become adsorbed to bottle walls, which can decrease the ultimate ^234^Th signal by as much as 25% if samples are not acidified within 6 h of collection [25]. Acidification also assists in the breakdown of organic matter. After acidification and ^230^Th addition, samples are allowed to equilibrate for at least 6 h, but may be stored for up to several days prior to additional processing. Note that the 6-h equilibration time is less than the 8–12 h suggested in initial method development [25, 30], but no significant difference is found in the ^234^Th signal or ^230^Th yield from equilibration time reduction [31]. Using a smaller volume not only reduces filtration time (~ 9 h average for 4 L samples, ~ 2 h average for 2 L samples in offshore water), but it is also advantageous when multiple groups are sharing water collected from a standard 10–12 L Niskin bottle. Updated recommendations for this step are using 2 L of unfiltered seawater, and decreasing equilibration time to 6 h.

#### Test of the efficacy of ^230^Th as a Tracer of ^234^Th

**Fig. 2:**
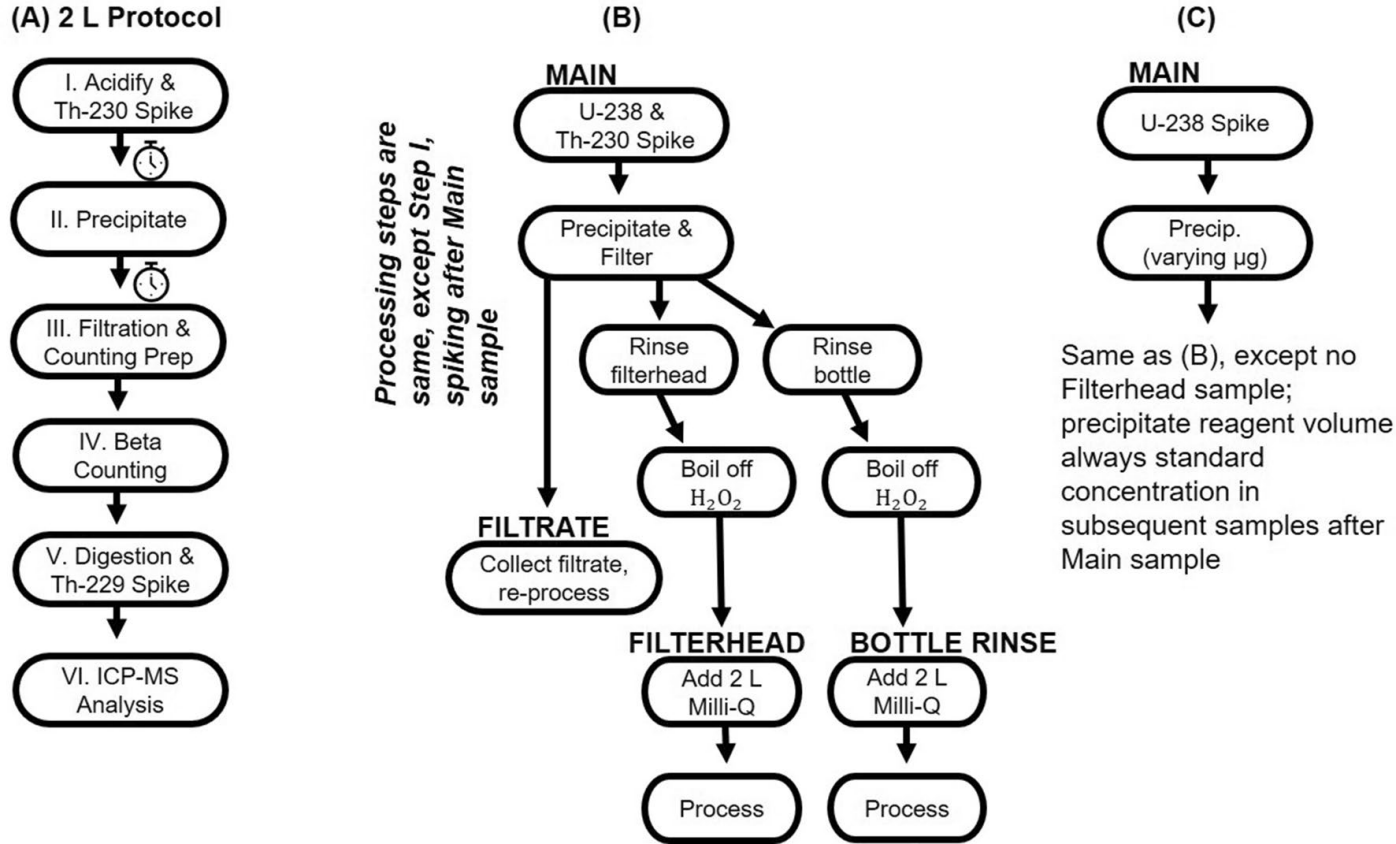
Schematic of the updated 2 L protocol presented in this manuscript (**a**), as well as overview of the protocol validation experiment (**b**), and overview of the experiment varying precipitate reagent amounts (**c**), introduced in “Step II: Formation of MnO_2_ Precipitate”

In order to assess the efficacy of the 2 L protocol and ^230^Th recovery method detailed above, ten 2 L seawater samples collected from the Woods Hole Oceanographic Institution (WHOI) dock (41.52°, -70.67°) were spiked with a high activity SPEX CertiPrep ^238^U solution (~ 100 dpm L^−1^) and ^230^Th solution. Each water sample had separate subsamples collected during processing – the Main, Filtrate, Bottle Rinse, and Filterhead Rinse – which allowed for tracking of both ^234^Th (in equilibrium with ^238^U), and ^230^Th tracer through the Main sample and the 3 potential components of sample loss (Fig. 2b). Ideally, the ^230^Th yield monitor tracks ^234^Th through all potential losses. The Main samples refer to the thorium species initially scavenged by the MnO_2_ precipitate and captured by the filter. While filtering the Main samples, the filtrate was collected, and the Filtrate samples refer to the thorium species scavenged by measuring thorium in the collected filtrate. The Bottle Rinse samples refer to the thorium scavenged on the bottle walls. Bottles from the original filtration are rinsed with an 8 M HNO_3_/10% H_2_O_2_ solution, H_2_O_2_ is then removed via boiling, and the remaining solution is added to 2 L of Milli-Q water and treated as a new sample. Similarly, the filterheads are rinsed and a new subsample is collected—the Filterhead sample.

**Fig. 3:**
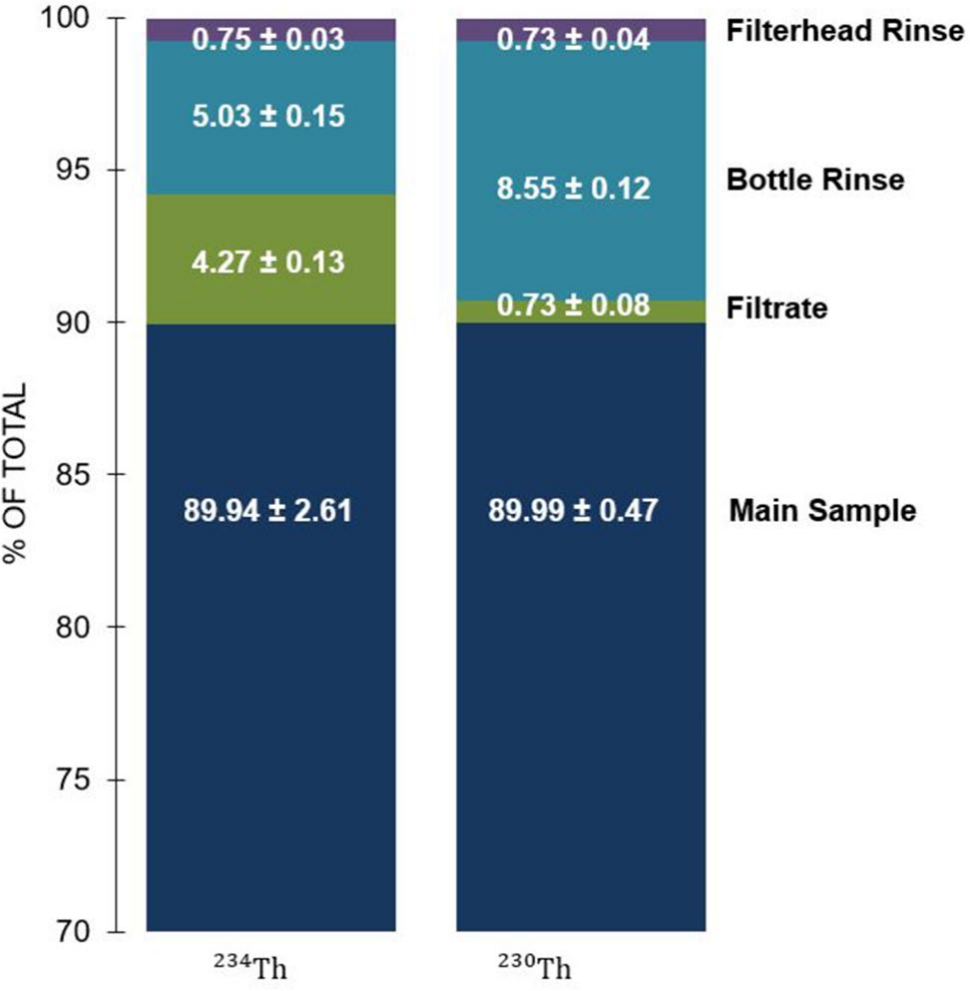
Percentages of total signal represented by each of the four sample components: Main, Filtrate, Bottle Rinse, and Filterhead Rinse. Columns represent average values (*n* = 9) from both ^234^Th and ^230^Th analysis

The resulting 40 samples were analyzed for both ^234^Th and ^230^Th specific activities, resulting in 80 total data points. Thorium isotopes for each sample were compared to ensure that they were tracking each other after equilibration in all four components of the original sample. The data are averaged for each sample component and summarized in Fig. 3 with the exception of data from one sample likely compromised by an incorrectly mounted or torn filter. The key result is that ^234^Th and ^230^Th in the Main sample tracked each other remarkably well, with an identical yield of 90% ± 3 and 90% ± 1 (see Fig. 3 for significant figures), respectively, of the thorium specific activities added to the original sample (*n* = 9).

The Bottle Rinse and Filtrate data, however, suggests that ^234^Th (experimentally added here by ingrowth from ^238^U) and ^230^Th (always added as a thorium tracer in acidic solution) do behave differently, with more of the ^234^Th losses associated with the filtrate and more ^230^Th losses to the bottle walls. Thorium speciation has been studied in seawater and reported in publications such as Santschi et al. [1], Somayajulu and Goldberg [32], and Hirose and Sagimura [33]. While it is clear that thorium isotopes in general have varying binding coefficients to different types of matter [1], the distinction between binding coefficients of ^234^Th and ^230^Th on the same material is less clear. It has been shown that different thorium nuclides (e.g., ^228^Th, ^230^Th, ^232^Th) can have varying binding strengths to the same organic material, resulting in different ^230^Th /^232^Th ratios in different minerals from the same sediment samples [32, 33]. A possible explanation for the lack of consistent tracking in Filtrate and Bottle Rinse data is that ^230^Th, added in pure ionic form, is more susceptible to bottle wall adsorption, while ^234^Th, produced here from added ^238^U more readily forms complexes with low molecular weight dissolved organic matter that is able to pass through the 1 µm QMA filter pores. In other words, differences in ^234^Th and ^230^Th activities during sample processing are driven by nuclide source versus mass fractionation. These differences in ^234^Th and ^230^Th behavior in the Bottle Rinse and Filtrate samples present an interesting area for further study.

The finding of identical recovery on the Main sample MnO_2_ precipitate validates the use of ^230^Th as a tracer of naturally occurring ^234^Th in the 2 L method.

### Step II: Formation of MnO_2_ precipitate

After 6 h of equilibration, the pH is increased to between 8.3 and 8.5 using ~ 3 mL concentrated ammonium hydroxide (NH_4_OH) in order to allow for precipitate formation. Creating the MnO_2_ precipitate includes the addition of 100 µL each of first a KMnO_4_ solution (7.5 g L^−1^ H_2_O) and then a MnCl_2_ solution (20 g L^−1^ H_2_O), shaking well between each reagent addition. Samples are allowed to sit for 8 h before filtration. Previous publications have recommended longer wait times between precipitation and filtration [e.g., 26], but 8 h has been found to be sufficient based upon consistent and high chemical yields, while maintaining reasonable filtration times, based on analysis of the authors’ previous thorium datasets [e.g., 31]. The updated recommendation for this step is decreasing precipitation holding time to 8 h.

#### Test for optimization of the MnO_2_ precipitation

Precipitation reagents of 100 µL (at the concentrations of the solutions described previously, this is 750 µg KMnO_4_ and 2000 µg MnCl_2_) are used, which are 1/20th of the amount originally recommended by Rutgers van der Loeff and Moore [24] for 20 L samples [24]. We tested whether the amount of reagents added may be decreased even further, with a goal of increasing sample throughput by reducing filtration times, and potentially increasing counting efficiency by reducing adsorption of beta emissions due to a thinner MnO_2_ precipitate. To determine if 100 µL (hereafter referred to in µg units) reagent solutions amounts are optimal, ten 2 L samples of coastal water collected from the WHOI dock were filtered through a 0.45 µm polyethersulfone (PES) filter, spiked with a SPEX CertiPrep ^238^U solution (~ 150 dpm L^−1^) immediately after collection, and then acidified. Similar to the experiments described in Step I, sufficient ^238^U was added such that the amount of ^238^U dominates any naturally occurring ^238^U (and thus ^234^Th), and samples were processed to account for components of loss (protocol described in Fig. 2c).

Each original ^238^U sample ultimately consisted of 3 fractions: Main, Filtrate, and Bottle Rinse samples. No Filterhead sample was collected in this experiment. Varying amounts of both reagents, KMnO_4_ and MnCl_2_ solutions, were tested in replicated samples: 187.5 µg KMnO_4_ & 500 µg MnCl_2_ (*n* = 3), 375 & 1000 µg (*n* = 2), 750 & 2000 µg (*n* = 3), and 1500 & 4000 µg (*n* = 2). Note that the standard amount used in the protocol detailed above is 750 & 2000 µg KMnO_2_ and MnCl_2_, respectively. A larger amount, 1500 & 4000 µg, was tested to confirm that the overall MnO_2_ precipitate scavenging efficiency would not be improved by increasing the amount of reagents used.

**Fig. 4:**
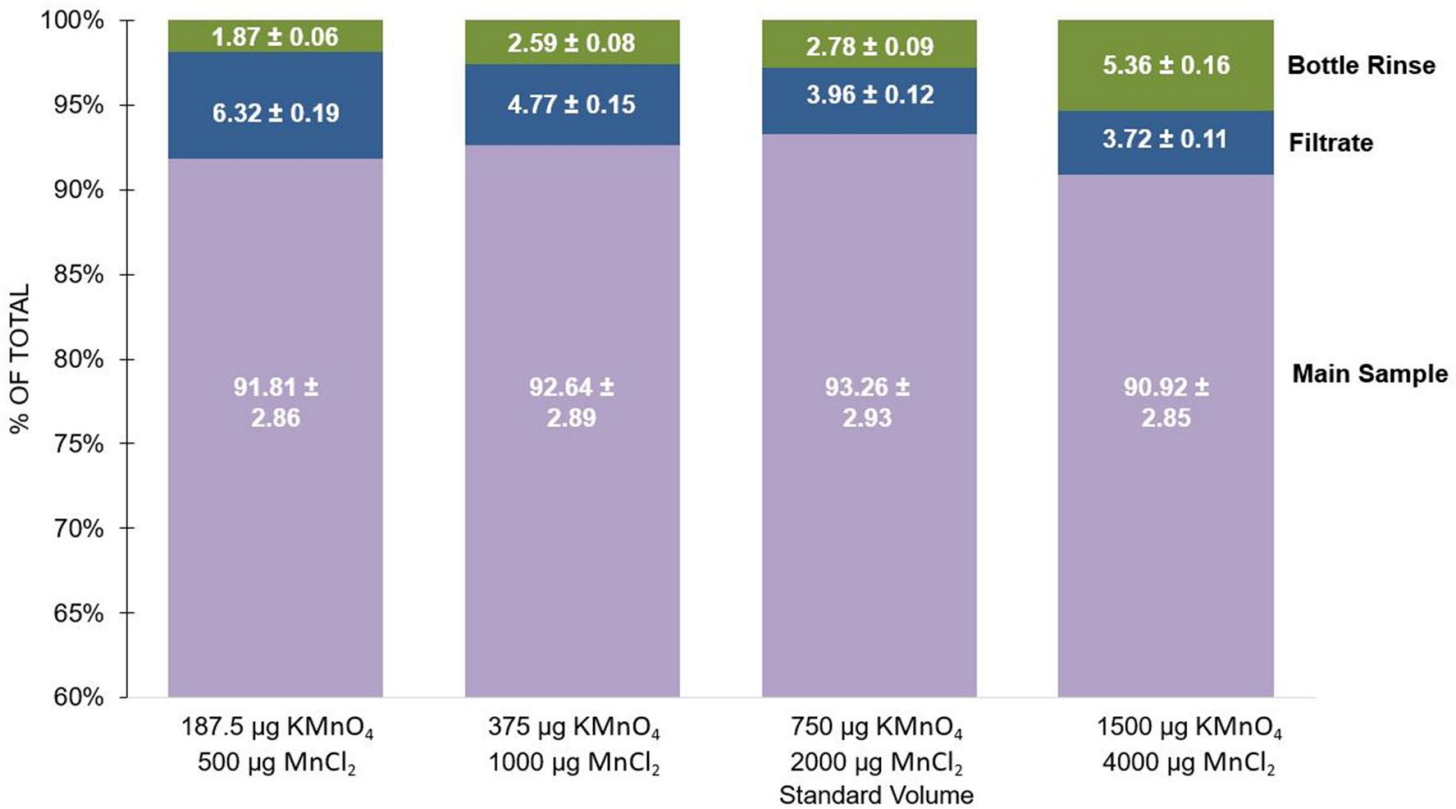
Percentages of total ^234^Th signal represented by each of the three sample components: Main, Filtrate, and Bottle Rinse. Columns are average data values for groups of samples using a range of MnO_2_ reagent amounts

There is no significant difference between the average ^234^Th recovery in the Main sample across the range of reagent amounts added (Fig. 4), with recoveries ranging from 91% ± 3 to 93% ± 3. In contrast, average ^234^Th within the Filtrate decreases with increasing reagent amount, by close to 50% when increasing from 187.5 & 500 µg to the standard amount and 1500 & 4000 µg. This is likely because the precipitate formed by higher amounts of reagents leads to either more efficient co-precipitation, and/or a thicker layer of precipitate on the filter, which helps increase filtration efficiency. An eightfold increase in reagent amount leads to a > 20% increase in ^234^Th activity associated with the Bottle Rinse. At the same time, however, the highest amount (1500 & 4000 µg) of precipitate reagents formed quantitatively more precipitate, which was observed to adhere to bottle walls. Variations in Filtrate and Bottle Rinse ^234^Th activities balanced, such that the relative activity in the Main sample, across all amounts of reagent tested, remained the same across all volumes tested.

Doubling the reagent volume does not increase ^234^Th recovery. Reducing reagents decreases the amount of time needed to filter samples (average filter times: 187.5 & 500 µg = 0.14 h, 375 & 1000 µg = 1.13 h, 750 & 2000 µg = 7.4 h, 1500 & 4000 µg = 11.5 h), a significant bottle neck in processing. In a natural setting, however, the magnitude of detrital or biogenic particles may have the greatest impact on filtration times, hence longer filtration times may be unavoidable, for example in surface versus deep waters and coastal versus open ocean samples, etc. Interestingly, the absolute ^234^Th count rate also did not vary across reagent amount, suggesting that the amount of precipitate does not significantly impact detector efficiencies (Main sample counts varied by ≤ 2.5%). The sums of the total counts of the three components of each original sample (consisting of Main, Filtrate, and Bottle Rinse samples) vary by ≤ 4.0%. Similar tests should include samples from other types of environments (e.g., open ocean). These results confirm that an eightfold reduction in precipitate amount via a decrease in reagent volume, effectively scavenges ^234^Th from 2 L samples.

### Step III: Sample filtration

Samples are filtered using custom filterheads, which thread directly onto a narrow-mouth 2 L bottle, hold a filter, and attach to a vacuum manifold. These filter holders are designed to support 25 mm diameter filters. Bottles are then inverted for filtration. Care should be taken to record filtration times since the time difference between the mid-point of filtration and sample collection is needed to correct for in-growth of ^234^Th from ^238^U within the sample solution in the collection bottle. Minimizing the time between collection and filtration reduces the correction for ^234^Th ingrowth from ^238^U. Recorded filtration times also assist in quality control, as samples that filter faster than others are likely candidates for low ^230^Th recoveries due to a cracked or misaligned filter, imperfect addition of precipitate reagents, or improper pH adjustment. Once filtration is complete, filters are rinsed inside of the filter head 2–3 times with a small amount of pH 9 water (created from NH_4_OH drops added to distilled water) to ensure that no precipitate is lost to the filterhead walls. Lower pH water would dissolve the precipitate and is thus not recommended. Filters are then placed in a petri dish and dried at 50–60 °C for 1–4 h.

It has been demonstrated that heating 2–5 L samples post-precipitation in a water bath (> 80 °C) and then cooling, a process that takes 3–5 h, can decrease filtration time to as low as 30 min [34]. However, reducing sample volumes from 4 to 2 L decreases average filtration time of offshore waters from ~ 9 to ~ 2 h. Coastal water samples, with higher organic matter content, still only have filter times of ~ 5 h. Therefore, reducing sample volumes, without the added step of sample heating, is an effective and less labor-intensive method to decrease sample filtration time.

#### Test of potential artifacts due to airborne radioactivity

Radioactive beta emitting isotopes occur naturally in the atmosphere throughout the world. An example pertinent to the ^238^U decay series is the emission of ^222^Rn from soils, rocks and building materials such as concrete, which decays to ^214^Bi in a matter of days. Further, more relevant to at-sea work, ^222^Rn is exhaled from ocean surface waters into the air where it decays to ^214^Bi. During filtration, it is possible that air flows through the MnO_2_ precipitate as it sits unattended and for variable amounts of time in the filtration rack once the sample is done filtering. This air may contain atmospheric radionuclides with a high enough beta decay energy to be detected.

To assess the potential for this process to impact the background count rate, 2 L samples of unspiked Milli-Q water were acidified and precipitated with manganese reagents according to the 2 L protocol presented here. Samples were kept in place with continued air flow being drawn through the filter for 0, 1, 2, 4, 6, and 8 h (*n* = 5 replicated for each set) and then beta counted.

**Fig. 5:**
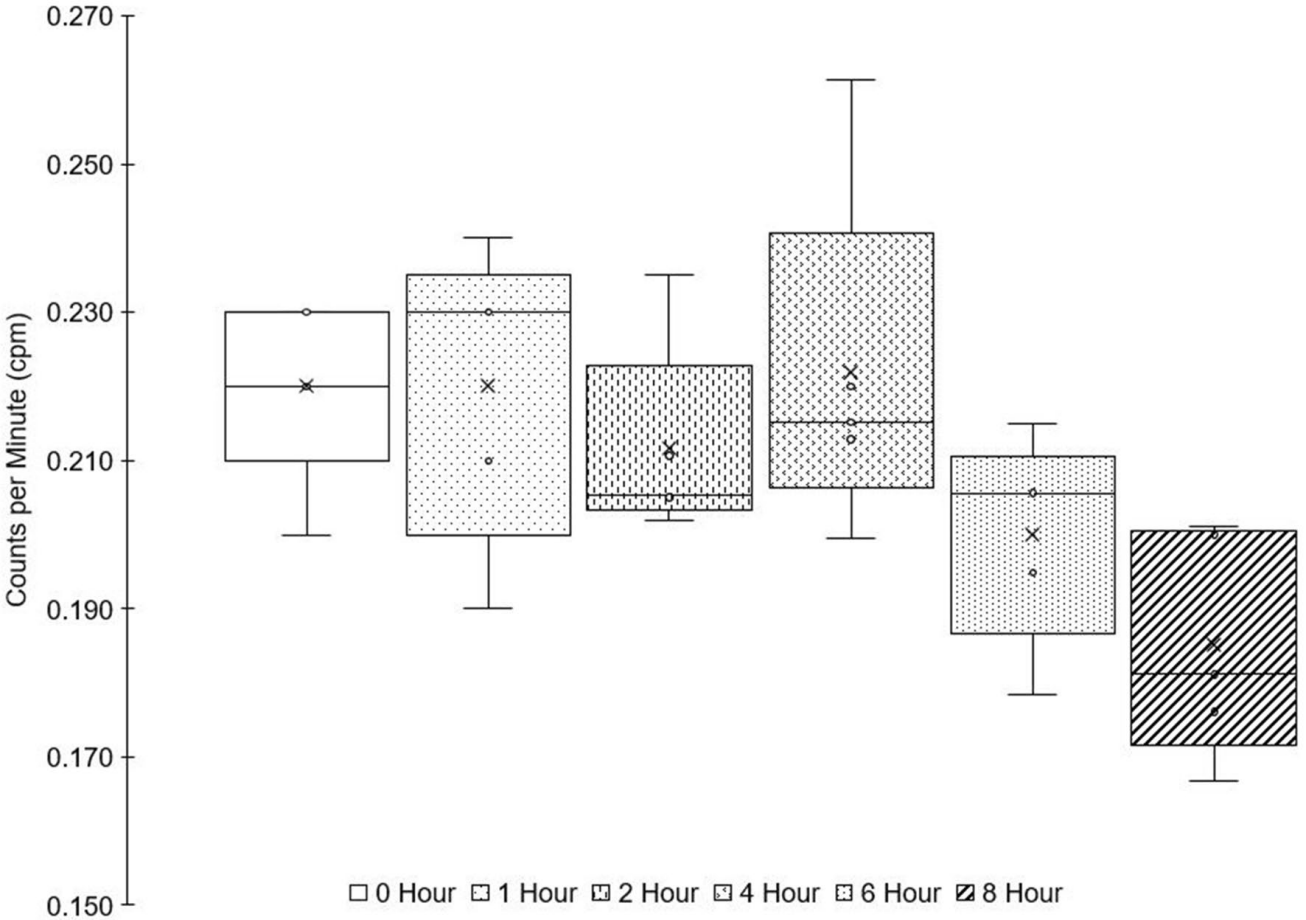
Results of un-spiked Milli-Q water samples over-filtered by varying times. Groups of 5 samples were over-filtered by the same number of hours

No statistically significant trend was found with filtration time, indicating that ambient beta emitting isotope signals are minimal (Fig. 5). It is important to note that this experiment was done on land, in a concrete building that is assumed to have a higher ambient level of ^222^Rn than at sea. Thus, airborne radioactivity contamination is likely even more negligible at sea than it has proven to be in this experiment.

### Step IV: Beta counting

*Initial counting*: Prior to beta counting, dry filters are mounted on custom beta mounts for use in low-level beta counters (DTU Risø Laboratories), covered with one layer of mylar and two layers of aluminum foil (2.24 g cm^−3^ per foil). The MnO_2_ precipitate can carry several non-thorium radionuclides present in seawater. The aluminum foil is thus added to block low-energy beta emitters, such as the isotopes of radium (e.g., ^226^Ra) and their short-lived daughters, reducing their contributions to the beta count rate [15]. As such, this foil also blocks the low energy level beta decay of ^234^Th itself (reaching no more than 0.19 MeV). As a result, the signal that beta counters detect is primarily the beta decay of ^234m^Pa, which, given its 1.2-min half-life, is in secular equilibrium with ^234^Th. With ~ 90% of the ^234m^Pa beta emission energy reaching 2.31 MeV, ^234m^Pa penetrates the double aluminum foil layer that many of the downstream ^238^U series daughter beta emitters cannot. The most likely contributor to additional activity is ^214^Bi, with multiple beta decays having an energy above 1.0 MeV, including ~ 19% of its beta emission energy reaching 3.26 MeV. This is one of the main reasons why ^234^Th samples must be counted more than 6 half-lives after initial collection in order to quantify the non-^234^Th signal naturally found in seawater and recovered by the same Mn precipitate that carries ^234^Th.

Once samples are prepared for counting, they are inserted into the 5 place anti-coincidence, low-level Risø beta counters. Each beta counter should be surrounded by 8 cm of lead to minimize natural background activity (average of 0.26 counts per minute (cpm) at sea, 0.21 cpm in lab). Individual detector background count rates should be determined wherever counters are used. For the Risø counters, the two outermost detectors (1 and 5) have a 0.05 cpm increase in background count rate due to comparative lack of shielding and the configuration of the Risø anti-coincidence technology.

When beta counting, the order of samples is randomized such that systematic biases are minimized. For example, in depth profiles, the same detector is not always used for a single depth. As soon as possible after samples are prepared, they are counted until they reach < 3% one sigma counting uncertainty (error = 1/$\surd counts$). With an initial activity around 2 dpm L^−1^, and an average 40% detector efficiency, count rates typically range between 2 and 3 cpm, or ~ 1000 to 1500 counts in 8 h. A second beta count is completed within a few days to a week after. The second count is not essential, but it is useful as a quality control, if time and counting ability allows. First and second counts differing by > 0.2 dpm L^−1^ is often indicative of spreadsheet or data entry errors, or in some cases detector malfunctions. Previous publications have called for as many as 6 recounts within a few half-lives after collection and using the calculated ^234^Th decay to separate initial count rates from background activities [14, 32]. This is unnecessary; one to two counts when first collected, and one final recount suffice to obtain an accurate dataset. All counts are then corrected for ^234^Th in-growth from ^238^U between sampling and filtration, and ^234^Th decay since initial collection.

*Final count:* All ^234^Th samples must be recounted after at least 6 half-lives (144 days) to account for background beta signals. Background count rates average approximately 0.50 cpm (e.g., 0.48 ± 0.24 cpm, *n* = 950; [31]). After 6 half-lives, only ~ 1.6% of the initial ^234^Th activity remains, or ~ 0.03 to 0.05 cpm for an average initial count rate of 2–3 cpm. Aside from a small residual ^234^Th signal, the background consists of primarily two components. Approximately 0.20 cpm is natural detector background. The remaining ~ 0.25 to 0.30 cpm is hypothesized to be caused by non-^234^Th but high energy beta emitters present in seawater that are also scavenged by the MnO_2_ precipitate (See Step II).

As mentioned previously, there are several other beta emitters in the ocean, with some of the most abundant coming from the ^238^U decay series [27]. The most likely non-^234^Th signal in seawater that is collected by the MnO_2_ precipitate is from ^214^Bi, a product of ^226^Ra decay. Beta decay of ^214^Bi is of sufficient energy to penetrate the foil filter cover as it emits several beta decays with energies > 1 MeV. Radium-226 has low activities in seawater (0.07–0.34 dpm kg^−1^), but it is also efficiently scavenged along with thorium by the MnO_2_ precipitate. If it is assumed that ^214^Bi is in secular equilibrium with ^226^Ra (given the short half-life of the intermediate decay product, ^222^Rn (*t*_1/2_ = 3.8 days)) and 100% recovery of ^226^Ra by the MnO_2_ precipitate, the amount of ^226^Ra on the MnO_2_ filter, and hence ^214^Bi, would be equivalent to 0.14–0.68 dpm per 2 L sample. While the exact ^214^Bi counting efficiency is unknown, it is unlikely to be higher than 50%, as the average efficiency for ^234^Th/^234m^Pa is ~ 40%. This would result in a beta count rate of 0.07 to 0.34 cpm. The observed non-^234^Th background count rate of 0.25–0.3 cpm is thus consistent with this range. It should also be noted that ^222^Rn is part of ^226^Ra decay chain, and losses of ^222^Rn gas could reduce the ^214^Bi signal by as much as 80% [25].

By halving the volume from 4 to 2 L, the non-^234^Th background signal is similarly reduced by half [25]. It is advised that final counts should be made on the same counter and detector that the original first counts were made on. However, since the final count is a small fraction of the original count rate, small variations in detector efficiencies and backgrounds do not impact final numbers significantly.

#### Test of sample orientation on counting efficiencies

**Fig. 6:**
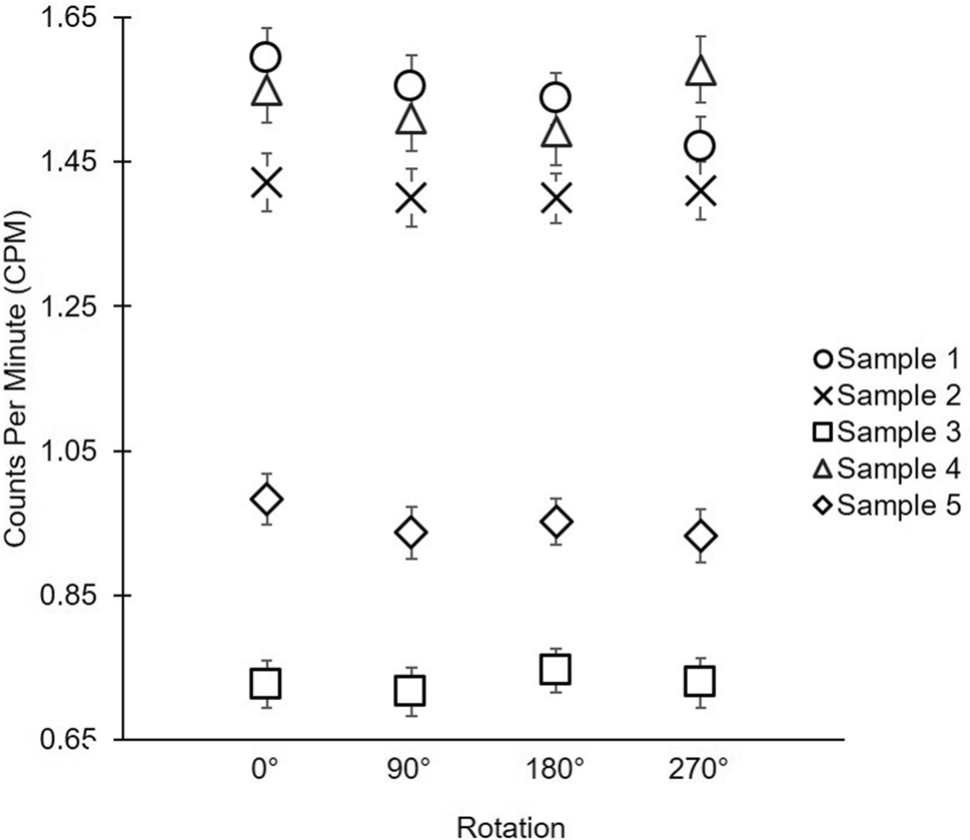
Five samples each rotated 90° 4 times. Variation would signal that sample orientation affects signal, but there is no significant difference within 95% confidence limit

To test whether imperfect sample mounting, i.e. a non-evenly distributed precipitate, might influence the count rates, we examined if sample orientation within the beta counter influences counting efficiency. To test this, five 2 L samples from the Bermuda Atlantic Time Series (BATS, 31.4º, -64.1º) were processed according to the 2 L protocol presented here, with count rates ranging from 0.7 to 1.5 cpm. Foil covers were marked to indicate direction within detector to ensure precise rotation. Each sample was then counted 4 times until errors reached < 3%, rotating exactly 90° between each count. The average variance of sample rotations is 0.03 cpm (Fig. 6). Based on this data, there is no significant difference in counts, within 95% confidence limit, when samples are rotated in the beta counter.

#### Investigation into detector stability

**Fig. 7:**
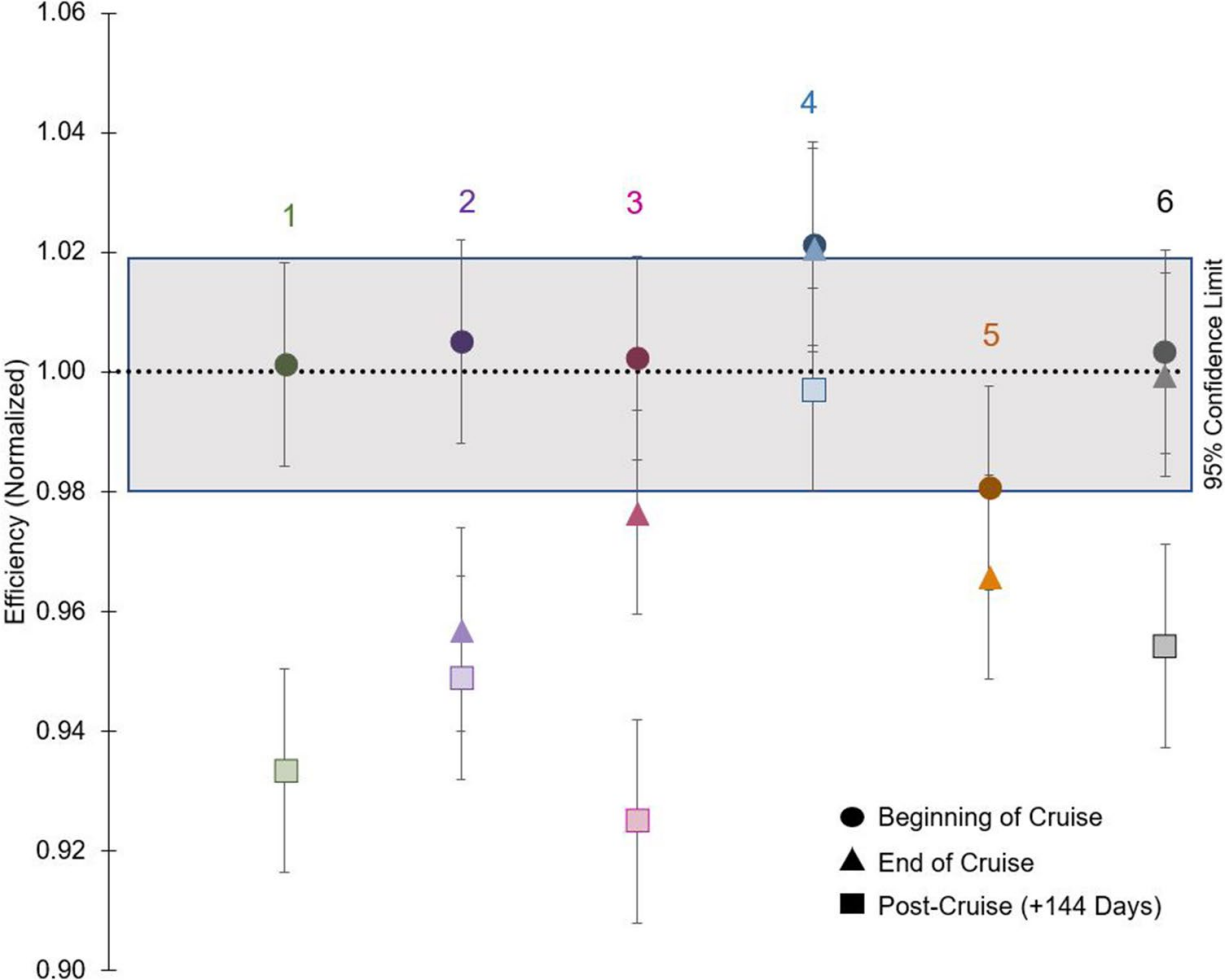
Standardized efficiency of 6 Risø counters used at-sea, with ^238^U standards counted 3 times. Data from the 2018 EXPORTS cruise [31]. Numbers above the data refer to Risø counter ID. Counter 1 is missing data from the end of the cruise, and 5 is missing post-cruise data

Cross-calibration between detectors and counters is essential to ensure that individual detector efficiencies are accounted for in the final results. At sea during the 2018 Export Processes in the Ocean from Remote Sensing (EXPORTS) field campaign each of the 6 Risø detectors was calibrated twice using uranium standards: once at the beginning and again at the end of the cruise, ~ 30 days apart. Detector efficiencies varied by < 2% in 24 of the 30 detectors, and by < 5% in the remaining detectors between the beginning and end of the cruise [31] (Fig. 7). When Risø counters were calibrated again ~ 6 months later on shore, they showed, on average, a 4% lower efficiency. Note that this does not impact data significantly as this correction to the final count rate (0.48 ± 0.24 cpm) is small. However, this data supports the recommendation that detector efficiencies should be reassessed between counter uses, especially at sea.

**Fig. 8:**
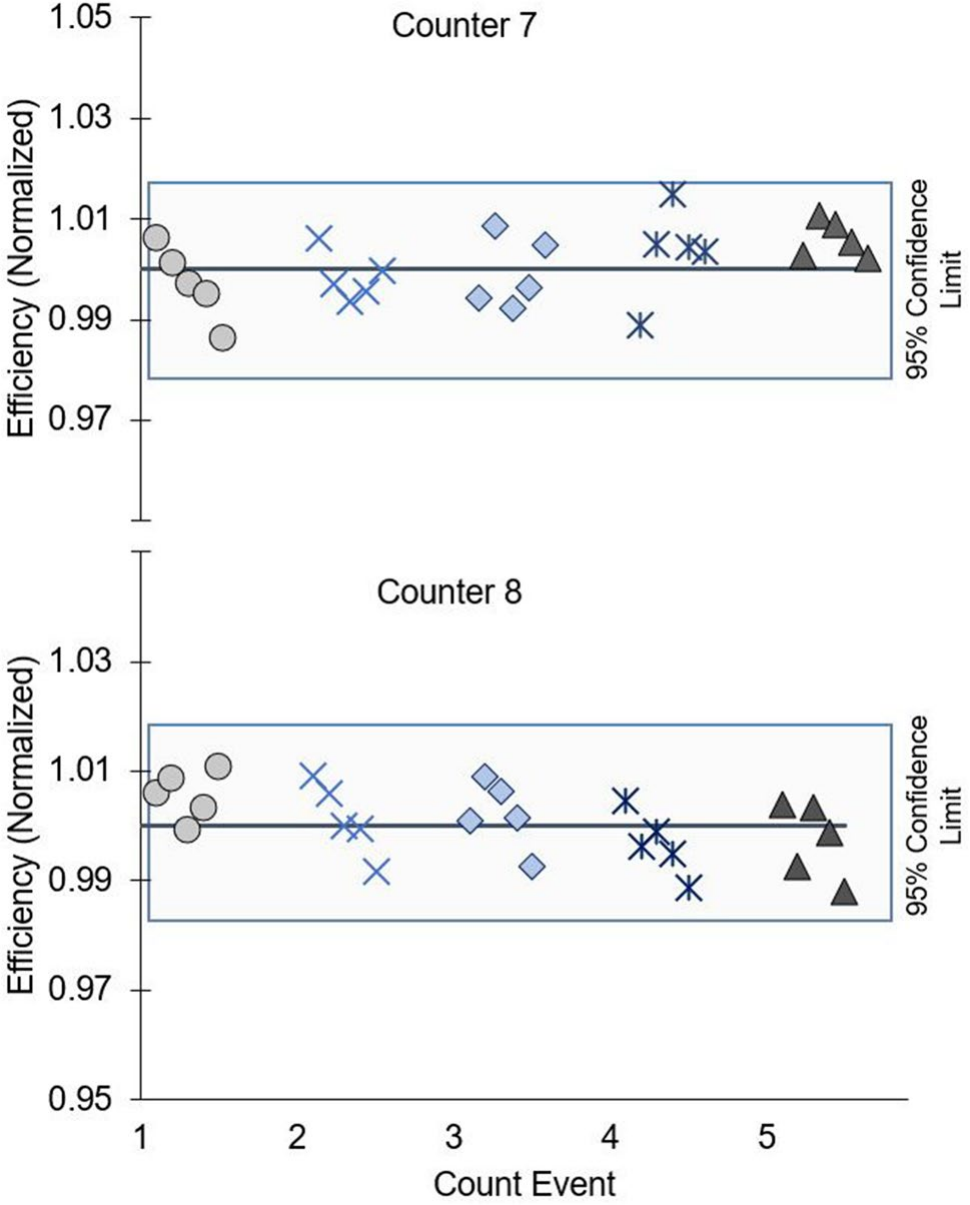
Standardized efficiency of 2 Risø counters used in-lab, with ^238^U standards counted 5 times. Data is shown by detector; count event is clustered by detector and staggered

The same type of Risø detectors used during the EXPORTS campaign were further assessed for efficiency variability on land. Less drift was observed between two different Risø detectors over a 60-day period when counted every 2 weeks (Fig. 8), with on-land efficiencies varying by < 1.8% among the 10 detectors and 50 ^238^U standard measurements. This higher variability in detector efficiency at-sea may be related to using a larger dataset with 6 counters of varying ages, and other factors such as the power supply at sea being less stable than in the lab.

Considering the possibility of variations in detector efficiency both at-sea and on land, it is recommended that ^238^U standards be counted more frequently than just at the beginning and end of a cruise. After an initial cross-calibration using 5 ^238^U standards rotated through each detector, individual detector efficiency drift should be monitored throughout a period of extended use. For the sake of counting time, rather than rotating through each of the 5 ^238^U standards per detector per counter multiple times, one ^238^U standard can be assigned to one detector and recounted on that detector multiple times throughout a period of extended use. Implementing this change would allow for tracking of individual detector efficiency drift.

### Step V: Sample digestion and Th recovery

Once the final beta count is completed, the sample is prepared for inductively coupled plasma mass spectrometry (ICP-MS) analysis in order to determine the ^230^Th spike recovery and correct for any missing ^234^Th lost during processing. As detailed in Pike et al. [26], an unmounted filter is placed in a 50 mL beaker, and the precipitate is dissolved upon the addition of 10 mL of an 8 M HNO_3_/10% H_2_O_2_ “recovery solution.” The beaker is immediately covered with a watch glass to prevent evaporation. Next, a 1 mL aliquot of ^229^Th (~ 70 dpm g^−1^) is added to track the amount of ^230^Th present that may have been lost due to MnO_2_ digestion and processing prior to ICP-MS analysis. The exact amount of ^229^Th added must be recorded in order to establish a ratio of atoms of ^230^Th to ^229^Th that should be present in a sample, although the ratio itself is arbitrary. The filters in solution are then sonicated for 20 min and allowed to sit overnight. Sitting overnight is a longer wait time than previous publications have used [e.g., 26], but reduces instances of clogging of ICP-MS lines.

#### Test of MnO_2_ precipitate digestion

Experiments were completed to quantify the effectiveness of digestion of the MnO_2_ precipitate to ensure that no ^234^Th remains on the filter post-digestion. This is important because the assumption is that the material that is beta counted is removed completely from the filter, and represents the chemical yield, as quantified by the ^230^Th yield monitor. If, for example, 10% of the thorium remained associated with the filter, then the chemical yield determined from ^230^Th analysis would be 10% lower than what it actually was when the filter was beta counted. Thorium-234 and ^230^Th are typically digested from the MnO_2_ precipitate filter with a recovery solution that uses strong acid and hydrogen peroxide, as mentioned above. Experiments were done with varying concentrations of the recovery solution used to digest the MnO_2_ precipitates after the initial beta count to determine optimal solution conditions. Based on safety and environmental concerns, the solutions tested were: 1 M HNO_3_/10% H_2_O_2_ (*n* = 3), 8 M HNO_3_/10% H_2_O_2_ (*n* = 4), and 15.9 M HNO_3_/10% H_2_O_2_ (*n* = 3). In this experiment, 20 L of surface seawater were collected off of the coast of New Jersey (~ 450 km offshore; 38.27°, − 70.02°) and spiked with SPEX CertiPrep ^238^U solution (~ 100 dpm L^−1^). The sample was then split into 10 different 2 L samples, which were processed and counted in accordance with the 2 L protocol detailed here. Samples were then sonicated for 20 min and allowed to sit overnight, covered with a watch glass. Filters were subsequently removed from the solution, rinsed gently with Milli-Q water, and allowed to dry on a clean watch glass. Filters were then remounted and recounted to quantify any remaining ^234^Th on the now visibly clean filter. This experiment was replicated with 10 samples using coastal water from the Woods Hole Oceanographic Institution (WHOI) dock (41.52°, − 70.67°) using only the 8 M HNO_3_/10% H_2_O_2_ solution to check if particle loads influence the efficiency of extraction at the 8 M HNO_3_ concentration.

Results from off the coast of New Jersey suggest that the 8 M HNO_3_/10% H_2_O_2_ solution is highly effective at digesting the MnO_2_ precipitate and removing ^234^Th from the filter, with ~ 1% of the original ^234^Th signal remaining. Using a 1 M HNO_3_/10% H_2_O_2_ solution (*n* = 3) resulted in 12% ± 7 remaining on the filter, a 8 M HNO_3_/10% H_2_O_2_ solution (*n* = 4) resulted in 1% ± 0.5, and 15.9 M HNO_3_/10% H_2_O_2_ solution (*n* = 3) resulted in 1% ± 0.1. Therefore, higher acid concentrations than 8 M do not need to be used. Using a 1 M HNO_3_ recovery solution is not recommended, as 12% ± 7 remained on the filter. However, in coastal samples from WHOI dock, 7% ± 1 (*n* = 10) of the original ^234^Th signal remained on the filter with an 8 M HNO_3_/10% H_2_O_2_ solution. These differences are likely to be due to increased organic matter content in coastal versus offshore waters. In order to apply this protocol to coastal environments, further research should be conducted to better elucidate the conditions required for complete thorium extraction from filters.

Based on these results, it is recommended that precipitate digestion solution molarity is not decreased from 8 to 1 M HNO_3_. Further tests must be run to determine if acid strengths between 1 and 8 M are effective.

### Step VI: Inductively coupled plasma mass spectrometry (ICP-MS) analysis

After digestion of the MnO_2_ precipitate, ^229^Th spiking, and sonication, a 0.5 mL aliquot of each sample is filtered through a 0.2 µm PES filter, into standard plastic 2 mL ICP-MS sample vials, then diluted with 1.5 mL Milli-Q water. Samples are then ready for ICP-MS analysis.

**Fig. 9:**
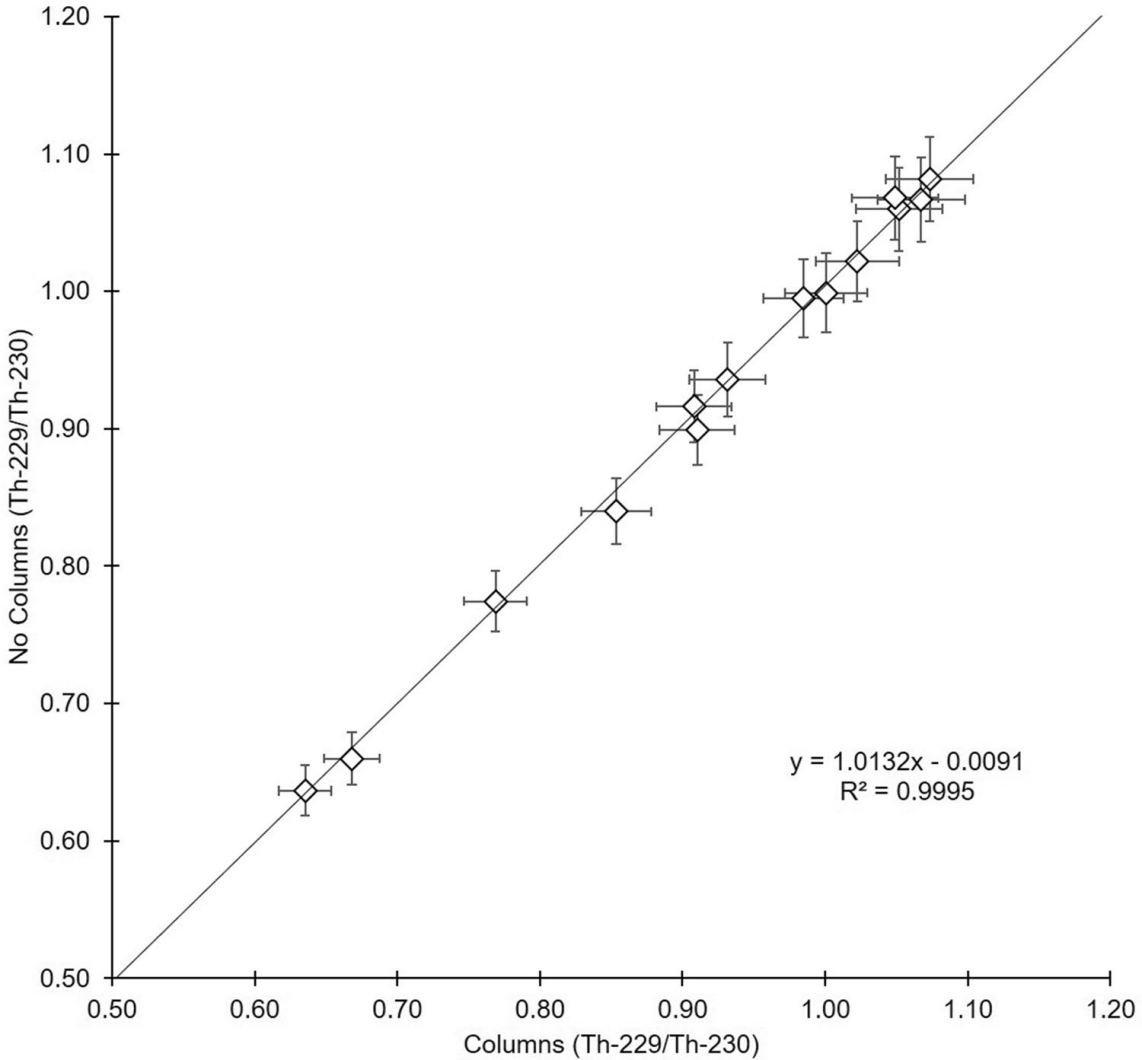
Comparison of samples analyzed using the standard anion-exchange column method versus the updated method omitting columns; adapted from data in [35]

This is a significant simplification of the protocol developed in Pike et al. [26] as it omits the need for anion-exchange column purification and the use of HF. These simplifications are primarily made possible by improvement in mass spectrometry technology. Anion-exchange column purification was originally necessary to remove manganese from the solution, which negatively impacted the performance of the mass spectrometer. Similarly, HF was added to the sample solution to aid in rinsing of the capillary lines. Puigcorbé [35] compared results obtained with and without using anion-exchange columns, and found no significant differences between the two methods (Fig. 9). Previous studies [e.g., 36–37] have omitted both anion-exchange columns and HF. This chemical recovery method was conducted on the 2018 EXPORTS campaign and resulted in average recoveries of 88 ± 9% (*n* = 950; [31]). These results are comparable to recoveries typically found in studies following the widely used method detailed in Pike et al. [26]. Foregoing anion-exchange column purification and omission of HF from the recovery solution allows for more expedient, lower cost sample analysis at less environmental and physical risk.

## Validation of the updated 2 L protocol

**Fig. 10:**
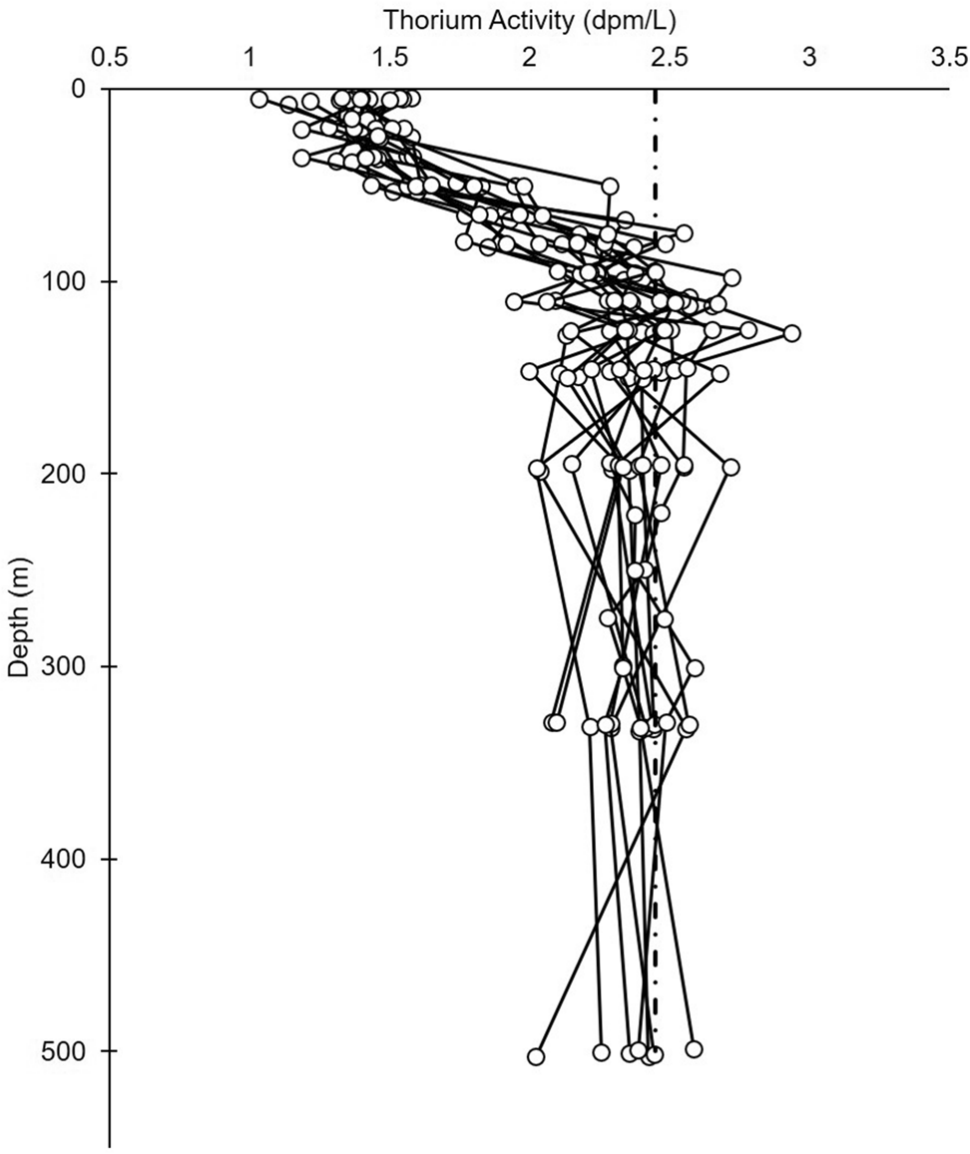
Example data of 15 out of 66 thorium profiles taken on a 2018 cruise to the NE Pacific (50°, -145°); data can be found at [31]. Round symbols are thorium-234 activities, while the dashed line indicates uranium-238 activity

The 2 L method presented here represents a significant improvement in the analysis of ^234^Th in seawater. The 2018 EXPORTS campaign used the 2 L method described here [31] and processed 950 samples over the course of 5 weeks at sea using a team of 4 people. Using these improved techniques, remarkably high resolution ^234^Th data can now be obtained, an example of which is shown in Fig. 10.

### Deep-sea water calibration

Calibration against deep water samples (ideally ≥ 2000 m, but above any bottom nepheloid layers) can confirm the accuracy of the 2 L protocol described here and is often used as an external check on detector calibration. At this depth, ^234^Th should be in equilibrium with ^238^U. During the 2018 EXPORTS cruise [31], 10 deep water samples were collected (2 casts of *n* = 5 samples; 3000 m), and analyzed via the 2 L protocol detailed here. ^238^U activity is calculated using its conservative relationship with salinity, as measured using the CTD and the following equation [38]:

1 $$^{238} {\text{U}} = 0.0786 \times {\text{S}}{-}0.315$$

In this case, mean and standard deviation of the 5 sample pairs for ^234^Th activities were 2.40 ± 0.14 and 2.42 ± 0.15 dpm L^−1^, which is identical within errors to the corresponding ^238^U activity of 2.41 dpm L^−1^ [31]. This correlation to within 0.5% between deep water sample ^234^Th activity and the calculated ^238^U activity suggests that the updated ^234^Th protocol yields high accuracy results, within counting statistics. In fact, the ultimate calibration of any ^234^Th method, including detectors, yield monitor and all calculations will be captured by this method. If one does not have access to deep ocean samples, an equilibrium check could similarly be conducted using aged seawater from any depth. Water stored for 6–10 ^234^Th half-lives will have returned to secular equilibrium.

## Conclusions

This paper has reviewed and presented an improved method for the analyses of ^234^Th in 2 L seawater samples. Experiments conducted here validate the efficacy of the method, and suggest further areas of possible methods development. The improved 2 L protocol is characterized by a decrease in sample volume, decreased waiting times between steps, and a significantly pared down recovery process. These changes have been proven to produce high-recovery samples with decreased overall processing time. Suggestions for future improvement are a decrease in precipitate reagents, and an increased attention to counter cross-calibration and detector drift.

Continued review of and improvement on the classic thorium analysis method is important for expanding the use of this integral tool in oceanography. Producing higher spatial, vertical and temporal resolution measurements expands the scientific community’s ability to understanding of the oceanic processes that control the fate of carbon associated with sinking particles and the biological carbon pump_._ In addition, new applications include studies of the fate and transport of organic pollutants and metals that are carried by these same particles.

## Data Availability

Data from EXPORTS can be found at: https://seabass.gsfc.nasa.gov/search/archive/WHOI/buesseler/EXPORTS/EXPORTSNP/archive.
